# A surgical case of aortic stenosis with recurrent gastrointestinal bleeding: Heyde syndrome

**DOI:** 10.1016/j.ijscr.2018.10.078

**Published:** 2018-11-10

**Authors:** Makoto Iijima, Nagomi Itoh, Ryota Murase, Yutaka Makino

**Affiliations:** Department of Cardiovascular Surgery, Oji General Hospital, Tomakomai, Hokkaido, Japan

**Keywords:** Case report, Aortic stenosis, Gastrointestinal bleeding, Heyde syndrome

## Abstract

**Introduction:**

The combination of aortic stenosis, acquired coagulopathy, and anemia due to gastrointestinal (GI) bleeding is described as Heyde syndrome.

**Presentation of case:**

We report a surgical case of a 77-year-old man who was admitted because of melena and exertional chest compression. GI endoscopy could not reveal the origin of the GI bleeding. Conservative therapy including fasting and transfusion improved the anemia. Echocardiography demonstrated severe aortic stenosis (AS) with a hypertrophied left ventricle. Hematologic examination by gel electrophoresis showed deficiency of high-molecular-weight multimers of von Willebrand factor (vWF), resulting in the diagnosis of Heyde syndrome. He electively underwent aortic valve replacement (AVR) with a bioprosthesis using standard cardiopulmonary bypass. In the perioperative period, the patient had no recurrent anemia. He was discharged without subjective symptoms on postoperative day 18. The 20-month follow-up was unremarkable, with no episode of recurrent GI bleeding.

**Discussion:**

Heyde syndrome is associated with acquired von Willebrand disease type IIA and AS. The true characteristic of von Willebrand disease type IIA is hemorrhagic diathesis caused by deficiency of high-molecular-weight multimers of vWF. Under the influence of high shear stress caused by AS, vWF is stretched and easily cleaved by vWF-cleaving protease. Consequently, it causes deficiency of high-molecular-weight multimers of vWF and primary hemostasis impairment. Therefore, the most effective treatment for Heyde syndrome is correction of AS. AVR can improve not only the hemodynamic status but also coagulopathy.

**Conclusion:**

The differential diagnosis of patients with recurrent GI bleeding with concurrent systolic murmur should include Heyde syndrome.

## Highlights

• We report successful surgical treatment of Heyde syndrome.
• The incidence of Heyde syndrome is expected to increase in aging population.
• Heyde syndrome should be considered in AS patients with gastrointestinal bleeding.
• Radical surgery should be performed regardless of gastrointestinal bleeding.

## 1 Introduction

Heyde syndrome, characterized by angiodysplastic gastrointestinal (GI) bleeding accompanied by aortic stenosis (AS), was first described by Edward C. Heyde in 1958 [1], and several similar cases have been reported since then. Although this condition is considered to develop in 20% of severe AS [2], it is not well known enough. With the incidence of AS rising in proportion to the increase in the elderly population, the incidence of Heyde syndrome is expected to increase in the future. Herein, we report a case of Heyde syndrome that was successfully treated by surgery.

This work has been reported in line with the SCARE criteria [3].

## 2 Presentation of case

**Fig. 1:**
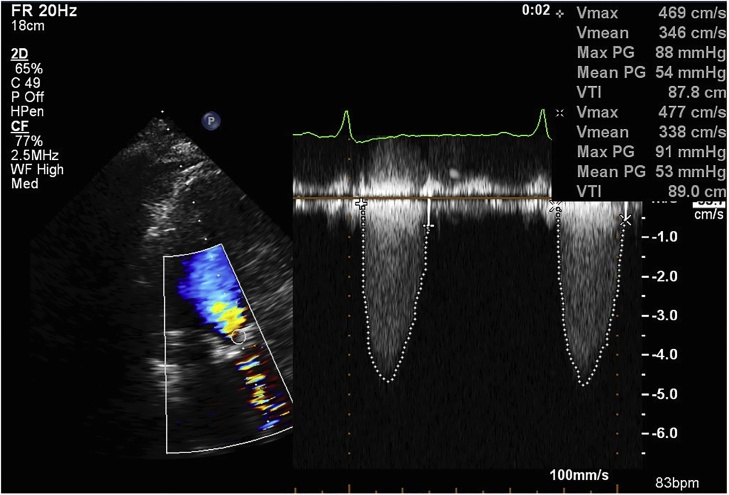
Preoperative echocardiography showing severe aortic stenosis.

**Fig. 2:**
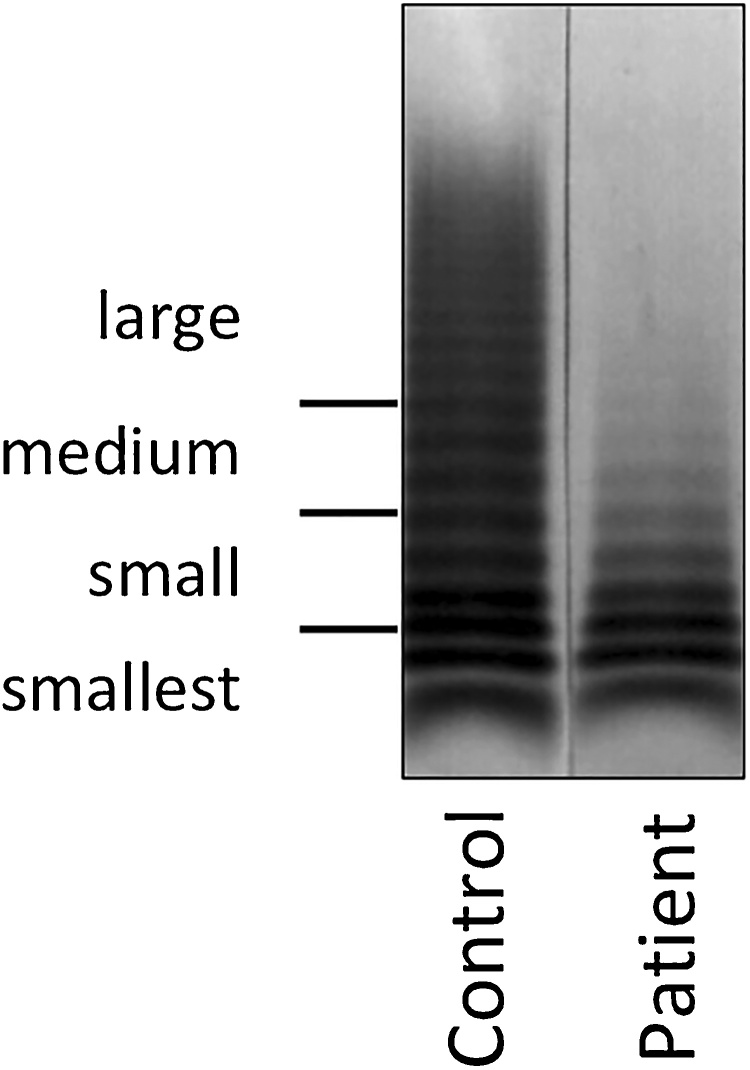
Multimer analysis of von Willebrand factor before aortic valve replacement.

A 77-year-old man was admitted to a local hospital because of melena and exertional chest compression. He had two hospitalization records in the past 1.5 years due to GI bleeding of unknown origin. Upon admission, the patient’s blood pressure was 105/56 mmHg, and his heart rate was 115 beats/min. Physical examination revealed palpebral conjunctival pallor and a Levine 4/6 systolic murmur at the second right intercostal space. Blood test results (hemoglobin, 5.5 g/dL; hematocrit, 16.2%) indicated advanced anemia, and fecal occult blood reaction was positive. GI endoscopy could not reveal the origin of GI bleeding. Conservative therapy including fasting and transfusion improved the anemia, and fecal occult blood reaction also became negative. Echocardiography showed severe AS, with a peak aortic pressure gradient of 88.0 mmHg and effective orifice area of 0.52 cm^2^ (Fig. 1). The left ventricular ejection fraction was 0.61. Although the serum level of von Willebrand factor (vWF) activity was in the normal lower limit of 51% (normal range 50–150%), further hematologic examination by gel electrophoresis showed deficiency of high-molecular-weight multimers of vWF (Fig. 2). Although the origin of GI bleeding was unknown, the patient was diagnosed with Heyde syndrome because of the presence of AS and deficiency of high-molecular-weight multimers of vWF. He was transferred to our institution for further treatment. Considering a low risk of intraoperative death (logistic EuroSCORE of 2.56% and risk of mortality from the Society of Thoracic Surgeons adult cardiac surgery risk score of 0.788%), the patient was considered a suitable candidate for surgical aortic valve replacement (SAVR).

**Fig. 3:**
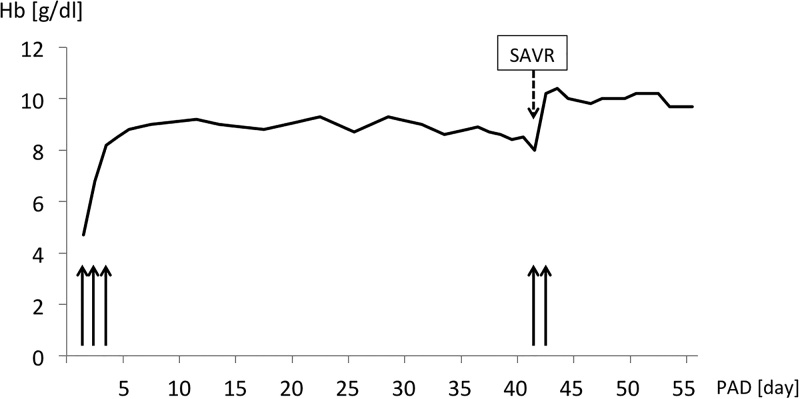
Clinical course and transition of hemoglobin levels. PAD, post-admission day; SAVR, surgical aortic valve replacement. Black arrow, red blood cell transfusion.

The patient underwent elective surgery. SAVR using a 21-mm bioprosthesis was performed with standard cardiopulmonary bypass (CPB), for which we selected unfractionated heparin anticoagulation. Aortic cross-clamp time was 71 min, and CPB time was 116 min. During the operation using heparin, there was no unexpected volume loss in which GI bleeding was considered. The postoperative course was uneventful. Under anticoagulant control of prothrombin time-international normalized ratio at 2.0–2.5, the patient did not develop recurrent anemia in the perioperative period (Fig. 3). The patient was discharged without subjective symptoms on postoperative day 18. The 20-month follow-up was unremarkable, with no episode of recurrent GI bleeding.

## 3 Discussion

Heyde syndrome is previously described as the association between AS and anemia due to GI bleeding from angiodysplasia [1]. By subsequent research, this disease is associated with acquired von Willebrand disease type IIA and AS [4]. Moreover, this case was diagnosed as Heyde syndrome, which developed in an elderly patient with AS because of deficiency of high-molecular-weight multimers of vWF, although angiodysplasia was not clearly recognized in the GI endoscopy.

Angiodysplasia is a submucosal vascular malformation. Lesions are multiple and frequently involve the cecum or ascending colon. It tends to occur in the elderly population; especially, the prevalence rate for people over 50 years old is 1.4–6.2% [5,6]. At least two hypotheses are included regarding how an angiodysplasia is formed: age-related degenerative change and intestinal hypoperfusion resulting from reduced pulse pressure that leads to stimulation of the sympathetic nervous system and consequently local vasodilation [7]. In the present case, colonic angiodysplasia was not detected by GI endoscopy. Tamura et al. reported that angiodysplasia was determined only at 33% in his series because of low rates of performance of endoscopic examination [8]. Angiodysplasia can develop in not only the stomach or colon but also in the small intestine [6]. Therefore, total GI examination, capsule endoscopy, as well as GI endoscopy should be considered to observe angiodysplasia in patients suspected of Heyde syndrome.

vWF is a platelet adhesion factor that is secreted into the blood as a homo-multimer [9]. The platelet aggregation activity becomes stronger as the molecular weight of the multimer increases. The true characteristic of von Willebrand disease type IIA is hemorrhagic diathesis caused by deficiency of high-molecular-weight multimers of vWF. Under the influence of high shear stress caused by the stenotic aortic valve, vWF is stretched and easily cleaved by vWF-cleaving protease, known as a disintegrin and metalloproteinase with a thrombospondin type 1 motif, member 13 [4]. Consequently, it causes deficiency of high-molecular-weight multimers of vWF and primary hemostasis impairment. In the present case, the preoperative serum level of vWF activity was normal. However, Vincentelli et al. reported that common coagulation-related tests including vWF often indicated normal results in von Willebrand disease type IIA [10]. For its diagnosis, vWF multimer analysis is indispensable.

For the treatment of Heyde syndrome, intestinal partial resection, coil embolization, and endoscopic cauterization have been previously performed, but a lot of recurrences were reported to date [11]. Given that the main cause of Heyde syndrome is a deficiency of high-molecular-weight multimers of vWF due to high shear stress, the most effective treatment is correction of AS, namely, SAVR or transcatheter aortic valve implantation [12]. Although there is concern about bleeding due to intraoperative anticoagulation using heparin, high-molecular-weight multimers of vWF are promptly corrected postoperatively [10]. In our case, the aortic valve area and peak pressure gradient through the aortic valve improved from 0.52 cm [2] to 1.42 cm^2^ and from 88.0 mmHg to 19.5 mmHg, respectively. vWF activity also improved from 51% to 142%; AVR had improved not only the hemodynamic status but also coagulopathy. Thompson et al. have reported that approximately 80% of cases of GI bleeding in patients with Heyde syndrome can be resolved by SAVR [13]. If there is an indication for a surgical procedure, SAVR or transcatheter aortic valve implantation should be actively considered.

## 4 Conclusion

We report a successful surgical case of Heyde syndrome. It is strongly anticipated that the number of patients with Heyde syndrome will increase in the future as patients with AS are expected to increase with aging. For patients with recurrent GI bleeding with concurrent systolic murmur, the differential diagnosis should include Heyde syndrome. The disease should be known to all. Physicians should not be hesitant about radical surgery because of gastrointestinal bleeding.

## Conflict of interest

None of the authors have any conflict of interest.

## Source of funding

None.

## Ethical approval

We obtained ethical certification with the approval number 2018-001 from ethic committee in our institution.

## Consent

Written informed consent was obtained from the patient for publication of this case report and accompanying images. A copy of the written consent is available for review by the Editor-in-Chief of this journal on request.

## Author contribution

Makoto Iijima: study design, data collections, data analysis, and manuscript preparation.

Nagomi Itoh: data collection and critical input.

Ryota Murase: data collection and critical input.

Yutaka Makino: data collection and critical input.

## Registration of research studies

Research Registry UIN: researchregistry4443.

## Guarantor

Makoto Iijima (First author).

Department of Cardiovascular Surgery, Oji General Hospital.

## Provenance and peer review

Not commissioned, externally peer reviewed.
